# Use and commercialization of *Podocnemis expansa *(Schweiger 1812) (Testudines: Podocnemididae) for medicinal purposes in two communities in North of Brazil

**DOI:** 10.1186/1746-4269-4-3

**Published:** 2008-01-21

**Authors:** Rômulo RN Alves, Gindomar G Santana

**Affiliations:** 1 Departamento de Biologia, Universidade Estadual da Paraíba, Av. das Baraúnas, 351/Campus Universitário, Bodocongó, 58109-753, Campina Grande-PB, Brazil; 2 Departamento de Sistemática e Ecologia, Universidade Federal da Paraíba, 58051-900 João Pessoa, PB, Brazil

## Abstract

**Background:**

Throughout Brazil a large number of people seek out reptiles for their meat, leather, ornamental value and supposed medicinal importance. However, there is a dearth of information on the use of reptiles in folk medicine. In North Brazil, the freshwater turtle, *Podocnemis expansa*, is one of the most frequently used species in traditional medicines. Many products derived from *P. expansa *are utilized in rural areas and also commercialized in outdoor markets as a cure or treatment for different diseases. Here we document the use and commercialization of *P. expansa *for medicinal purposes in the state of Pará, Northern Brazil.

**Methods:**

Data were gathered through interview-questionnaires, with some questions left open-ended. Information was collected in two localities in Pará State, North of Brazil. In the City of Belém, data was collected through interviews with 23 herbs or root sellers (13 men and 10 women). Attempts were made to interview all animal merchants in the markets visited. In fishing community of the Pesqueiro Beach, interviews were done with 41 inhabitants (23 men and 18 women) and during the first contacts with the local population, we attempted to identify local people with a specialized knowledge of medicinal animal usage.

**Results:**

*P. expansa *was traded for use in traditional medicines and cosmetics. Fat and egg shells were used to treat 16 different diseases. Turtle fat was the main product sold. The demand for these products is unknown. However, the use of this species in folk medicine might have a considerable impact on wild population, and this must be taken into account for the conservation and management of this species.

**Conclusion:**

Our results indicated that the use and commercialization of *P. expansa *products for medicinal purposes is common in North of Brazil. More studies regarding the use and commerce of Brazilian turtles are urgently needed in order to evaluate the real impact of such activities on natural populations. We hope that our findings about the trade and use of *P. expansa *in folk medicine will motivate further studies on the use of animals in folk medicine and its implications for conservation.

## Background

Traditional medicine has often used plants and animals, including turtles and tortoises, as healing elements or other kinds of treatment [1-3]. Discussions concerning the links between traditional medicine and biodiversity are therefore becoming imperative [4], particularly in view of the fact that folk medicine is the source of primary health care for 80 percent of the world's population. From a biological perspective, there is a need to increase our understanding of the biology and ecology of species commonly used as remedies, to better assess the impacts of harvesting pressure (for medicinal or other purposes) on their wild populations [5].

Reptiles are among the animals species most frequently used in traditional folk medicine, and their role in folk practices, as a major factor in the healing and/or prevention of illnesses, has been recorded in different socio-cultural contexts throughout the world [1-3,6-10]. In Brazil, reptiles have been exploited for many purposes such as food and medicine, pets, as a source of leather and other products [2,5,11-13]. The importance of medicinal animals for traditional communities from distinct socio-cultural-environmental landscapes in Brazil has been well documented [1,5,13]. Medicinal animals are widely used not only by rural communities, but also by people living in urban environments. The notable use of medicinal animals to alleviate and cure health problems and ailments in both rural and urban areas, points out the importance of this natural resource in the folk medicine and culture of these people [5]. Despite the intensive use and commercialization of medicinal animals in Brazil, documentation of zootherapeuticals practices remains scarce.

Among reptiles, turtles and tortoises are the most heavily exploited for human consumption [14]. Marine turtles are used in traditional medicine in many parts of the world [2,3]. Likewise, freshwater species of turtle are used and commercialized for medicinal purposes [8]. More than one-half of all freshwater tortoise and turtle species from southeast and eastern Asia are currently endangered or critically endangered, largely because of over-collection by the food and traditional medicine industries [14-17]. This highlights the importance of understanding such uses for the conservation of these species.

The turtle species that dwell in the world's rivers, play an important, but incompletely understood and largely unappreciated role both in the ecology of their respective ecosystems, and in the economy and sociology of human groups that subsist by using the rivers's resources [18]. Amazonia, with its 7 million square kilometers of rain forest, is traversed by numerous rivers and streams, which harbor a rich variety of aquatic life [19]. The human inhabitants of Amazonia depend on the immense biodiversity found there. Among the many species of animals occurring there, freshwater turtles are important commodities. River turtles (genus *Podocnemis*) play a vital role in the regional economy, and can play a key role in the ecology of the flood-plain ecosystem, particularly by consuming and dispersing seeds of some tree species [14].

In South America, six species of the *Podocnemis *genus are found: *Podocnemis vogli, P. lewyana, P. expansa, P. unifilis, P. sextuberculata*, and *P. erythrocephala*. From these species, the last four are found in Brazil [20].

*Podocnemis expansa *(Schweiger 1812) is known as "Tartaruga da Amazônia" by locals. It is one of the largest freshwater turtles and may weigh up to 55 kg [21]. This species is widely distributed in Amazonia [22] and thrives in black, clear and muddy waters and it is found in most rivers and associated lakes in Amazonia [21]. In spite of its wide distribution, it is endangered over much of its range due to centuries of reckless exploitation for its meat, fat and eggs [21,23,24]. Given its vulnerability, it has been the object of much concern and efforts have been made to conserve the species [25].

In Amazonian area *P. expansa *is frequently used in traditional medicine. Yet, there is a dearth of information about the use of this species in folk medicine. Here we document the use and commercialization of this species for medicinal purposes in the state of Pará, Northern Brazil. We hope that our findings about the trade and use of *P. expansa *in folk medicine will stimulate further studies on the use of animals in folk medicine and its implications for conservation.

## Materials and methods

Our study was carried out from September to November 2005 in Belém and Pesqueiro Beach, in Pará State, Brazil. Belém is the capital and the largest city in Pará (01°27'21"S and 48°30'16" W). Its population exceeds 1.3 million, making it the 10th largest city in Brazil. Its metropolitan area has approximately 2.01 million inhabitants. Belém has hundreds of outdoor markets and shops selling agricultural commodities, fish, and a wide range of the Amazonian flora and fauna. One of the regions' most famous outdoor markets is the "Ver-O-Peso" in the City of Belém, where fruit, fish, meat, herbs, medicinal products, bush meat, and handicrafts are sold [26].

The Pesqueiro Beach is located in the Municipality of Soure, on the eastern side of the Marajó Island, which is the largest fluvial island in the world. Soure encompasses an area of 3,528.7 km^2 ^and a population of 19,195 inhabitants. Cattle ranching is Soure's main economic activity, followed by subsistence fisheries and agriculture [13].

Data were gathered through interview-questionnaires, with some questions left open-ended [27]. In the City of Belém, this information was collected through interviews with 23 herbs or root sellers (13 men and 10 women). The sampling method was non-random, and the interviewees were pre-defined [28]. Attempts were made to interview all animal merchants in the markets visited. Yet, some interviews were cancelled, or the information gathered was minimal, because interviewees were reluctant to answer questions. These interviews with incomplete information were not considered in the analyses. In the Pesqueiro Beach, interviews were done with 41 inhabitants (23 men and 18 women) and during the first contacts with the local population, we attempted to identify local people with a specialized knowledge of medicinal animal use, following Davis and Wagner [29]. A specialist is defined as a person recognized by the community as having deep knowledge about the use of animals in manufacturing remedies and in promoting cures [1].

## Results and discussion

The traditional markets, where herb sellers are established, represent important sales points of the native exotic fauna of the Amazonian region. It is common to find specific locations in these markets where plants and animals are sold for medicinal purposes. Almeida and Albuquerque [30] believe that information on exotic and native flora and fauna obtained in these markets can serve as subsides for conservation strategies for commercialized resources. However, despite this importance, few ethnozoological studies have focused on herbal vendors in public markets and/or fairs.

**Figure 1:**
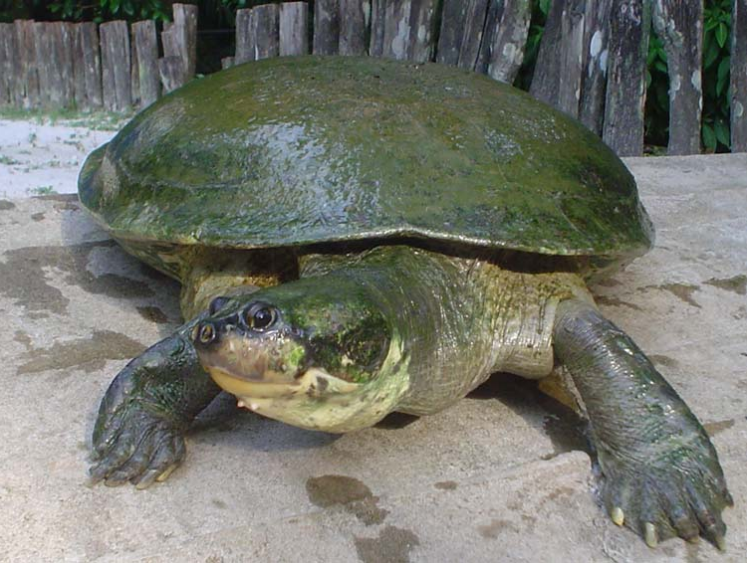
*Podocnemis expansa *known as "Tartaruga da Amazônia" (Photo: Rômulo Alves, 2006).

In the northern Brazilian Region, *P. expansa *(Fig. 1) is among the species most frequently used for medicinal purposes [7,13]. Medicinal products derived from *P. expansa *are utilized in rural areas and are commercialized in outdoor markets for treatment of diseases. In the City of Belém products derived from *P. expansa *have been traded for medicinal and cosmetic purposes. According to the interviewees (n = 23, 100%), products originating from "tortoises" are useful for treating several diseases including rheumatism, bleeding (wounds), arthritis, tumors, inflammation, cellulite, acne, blemishes and scars. Medicinal products of *P. expansa *can be used solely or in combination with other zootherapeuticals and phytotherapeuticals. The main reasons for popularity of animal-based remedies in surveyed areas were cultural aspects, perceived efficacy and economic accessibility.

**Figure 2:**
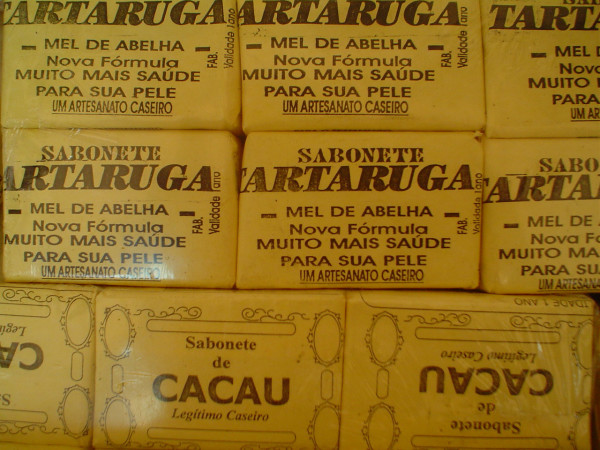
Soap derived of the fat at of the *P. expansa *being sold at the "Ver-O-Peso" market in Belém, among other medicinal folklore products (Photo: Rômulo Alves, 2006).

According to interviewees, fat is the most widely traded product from *P. expansa*. Fat is used to make a topical rub for wounds and aching muscles. This product can be taken orally for some illnesses. Soap is also produced from fat of *P. expansa *(Fig. 2) and has been used for treating acne and scars. All of the interviewed merchants said that turtles were sold and revealed that they obtained *P. expansa*, whole or in part, through sellers who periodically deliver these animals to them. The merchants also obtained turtles from people that come from rural areas and eventually capture turtles and other animals for trading. The therapeutic indications of *P. expansa *and domestic animals and plants species overlapped in many cases. This aspect opens a perspective of, when suitable, replacing the use of zootherapeuticals products derived of *P. expansa *with others in traditional medicine recipes, which in the long term could reduce pressure on the wild population of this species.

Although Brazilian legislation forbids commercial use of wild fauna (Article 1 Law 5,197 January 3, 1967 and Article 29 of Law 9,605 February 12, 1998), products and derivatives made from tortoises and many other animals are commonly illegally traded in Belém. Our observations are not unique and sale of turtle and tortoise products occurs in many other Brazilian cities [8,30-32]. This suggests that turtle and tortoises are used for commercial purposes throughout Brazil, and these activities may be expanding into urban areas.

**Figure 3:**
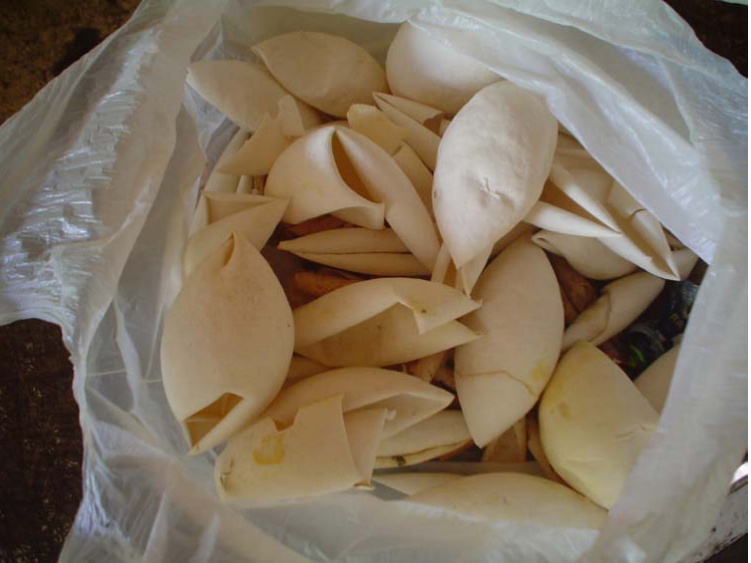
Eggs shells of *P. expansa *used as remedies at the Pesqueiro Beach, Marajó Island (Photo: Rômulo Alves, 2006).

In Pesqueiro Beach, we observed that fat from *P. expansa *was prescribed to treat 12 different diseases: inflammation, acne, tumor, boil, rheumatism, skin spots, backache, earache, arthrosis, arthritis and swelling. Additionally, eggs shells of *P. expansa *are used to treat pterigium (Fig. 3). Most respondents (n = 30, 73%) informed that the meat and eggs from these turtles are also consumed as food, what shows the importance this natural resource has as a complementary protein source in the communities surveyed. Klemens and Thorbjarnarson [14] also reported the exploitation of river turtles *(Podocnemis expansa *and *P. unifilis*) as food in Amazonia. A similar situation occurs in Bolivia, where *P. expansa *and *P. unifilis *are used as food and the trade of their meat and eggs is an important income for the peoples living in the forest [33]. Information such as those are important to be taken into account for plans of management and conservation of turtles. As pointed out by Alves and Rosa [1], the multiple uses of animals (including the medicinal aspects) and their impacts should be properly assessed and contextualized.

*P. expansa *is listed on the Convention on International Traded in Endangered Species (CITES) Appendix II, which legislates regulation and control of international trade for their protection [34]. Additionally, the species is rated "one" on the Tortoise and Freshwater Turtle Specialist Group 1989 action plan rating, a priority conservation-rating plan – indicating that it is "in trouble through over-exploitation for eggs and meat, and some are particularly vulnerable at colonial nesting sites". The concern with the conservation status of *P. expansa *populations and the potential of sustainable use of this natural resource have encouraged governmental and private organizations of Amazonian countries to dedicate efforts to protect and to restore its populations, as well as stimulating the establishment of captive production operations for commercial purposes [35].

Several threats are currently jeopardizing the long-term conservation of turtle populations around the world. *P. expansa *is an explicit case of the fragility of the turtle populations in the Amazonian region. The extreme and uncontrolled overexploitation of this giant river turtle has reduced its numbers to the point that its long-term survivorship is uncertain [36]. Habitat alteration, pollution, human exploitation, diseases and introduced species are directly implied in the decline of several turtles species around the world [37]. The use and trade of these animals for medicinal purposes can also have drastic consequences on their natural population [15-17]. Therefore, the impact the medicinal use of reptiles can have on the survival and conservation of wild turtles population should also be considered as important as other anthropogenic pressures [6].

The impacts of zootherapy on wild populations should be carefully assessed, mainly due to the general acceptance of zootherapeutical practices in Brazil [30]. Commercial harvest of *P. expansa *for medicinal purposes may impact their population, but the magnitude of these effects is poorly understood. Although the demand for these products is unknown, it may constitute an important issue for the conservation and management of *P. expansa *and other turtle species. Our findings highlight the need to further examine the interfaces between fisheries, illegal/unreported trade, regulations, and conservation in Brazil.

## Conclusion

Our results indicated that the use and commercialization of *P. expansa *products for medicinal purposes is common in North of Brazil. In Brazil, habitat destruction is one of the main threat to the natural populations of reptiles [38]. More studies about the use and commerce of Brazilian turtles are urgently needed in order to evaluate the real impact of such activities on natural populations. Medicinal species whose conservation status is a cause of concern should receive urgent attention, and aspects such as habitat loss/alteration should be discussed in connection with present and future use of these species in folk medicine [5,39].

Given the potential economic use of turtles, more attention should be focused on the development of sustainable use programs for these animals. It is essential that conservation and management programs involve the local communities who exploit natural resources. Community-based efforts such as these are limited by scarce funding, consistent and effective involvement of stakeholders, and political infighting. However, this is a strategy that has the most potential to redirect human behavior from unrestrained exploitation to sustainable use of a resource [33].

Turtle and tortoise management policies need to consider, among other things, the ecological roles of animals, biological limitations such as slow growth and late maturation, conservation status, human uses, institutional capacity to regulate wildlife use, as well as cultural and social impacts. If the need for conservation is to be accepted by people who make their livelihoods from wildlife or use wildlife as food and/or medicine, then care should be taken to avoid approaches with little or no social resonance i.e., that may be perceived as ideological or culturally imperialistic.

## Authors' contributions

RRNA – Ethnozoological data, writing of the manuscript, literature survey and interpretation; GGS – Analysis of taxonomic aspects, writing of the manuscript and literature survey and interpretation. All authors read and approved the final manuscript.
